# MicroRNA Dysregulation in the Spinal Cord following Traumatic Injury

**DOI:** 10.1371/journal.pone.0034534

**Published:** 2012-04-12

**Authors:** Mónica Yunta, Manuel Nieto-Díaz, Francisco J. Esteban, Marcos Caballero-López, Rosa Navarro-Ruíz, David Reigada, D. Wolfgang Pita-Thomas, Ángela del Águila, Teresa Muñoz-Galdeano, Rodrigo M. Maza

**Affiliations:** 1 Molecular Neuroprotection Group, Experimental Neurology Unit, Hospital Nacional de Parapléjicos (SESCAM), Toledo, Spain; 2 System Biology Unit, Experimental Biology Department, Faculty of Experimental and Health Sciences, Universidad de Jaén, Jaén, Spain; 3 Bascom Palmer Eye Institute, Miller School of Medicine, University of Miami, Miami, United States of America; Hertie Institute for Clinical Brain Research, University of Tuebingen, Germany

## Abstract

Spinal cord injury (SCI) triggers a multitude of pathophysiological events that are tightly regulated by the expression levels of specific genes. Recent studies suggest that changes in gene expression following neural injury can result from the dysregulation of microRNAs, short non-coding RNA molecules that repress the translation of target mRNA. To understand the mechanisms underlying gene alterations following SCI, we analyzed the microRNA expression patterns at different time points following rat spinal cord injury.

The microarray data reveal the induction of a specific microRNA expression pattern following moderate contusive SCI that is characterized by a marked increase in the number of down-regulated microRNAs, especially at 7 days after injury. MicroRNA downregulation is paralleled by mRNA upregulation, strongly suggesting that microRNAs regulate transcriptional changes following injury. Bioinformatic analyses indicate that changes in microRNA expression affect key processes in SCI physiopathology, including inflammation and apoptosis. MicroRNA expression changes appear to be influenced by an invasion of immune cells at the injury area and, more importantly, by changes in microRNA expression specific to spinal cord cells. Comparisons with previous data suggest that although microRNA expression patterns in the spinal cord are broadly similar among vertebrates, the results of studies assessing SCI are much less congruent and may depend on injury severity. The results of the present study demonstrate that moderate spinal cord injury induces an extended microRNA downregulation paralleled by an increase in mRNA expression that affects key processes in the pathophysiology of this injury.

## Introduction

Traumatic spinal cord injury SCI is characterized by a specific pathophysiological response that can be divided into three phases. The acute phase represents the initial trauma and affects the neural tissue directly, inducing a state of spinal shock. The acute phase is followed by a secondary phase that takes place over a time course of minutes to weeks after the injury and exacerbates the damage inflicted by the primary injury. The secondary phase comprises several interrelated damage processes that include vascular alterations, biochemical disturbances and cellular responses that lead to an inflammatory response and cell death. The chronic phase occurs between days to years after the trauma and is characterized by apoptosis, Wallerian degeneration and scarring that establishes functional impairment [1], [2]. Processes occurring after SCI are associated with altered gene expression patterns; there is a strong upregulation of genes related to inflammation and cell death along with a downregulation of genes involved in cell excitability and neurotransmission within the first hours after injury. Upregulation of the genes involved in inflammation and apoptosis persist during the first weeks, whereas genes regulating cytoskeletal arrangement, myelin ensheathment and synapsis show decreased expression, reflecting compromised tissue integrity. However, genes coding for angiogenic, neuritogenic and growth factors show increased expression, in an attempt to promote survival and regeneration [1]. This alteration of gene expression that is associated with processes triggered by SCI is thought to be accompanied by the post-transcriptional regulation of these modified gene networks. Among the known post-transcriptional regulators, microRNAs have recently attracted much attention due to their ability to inhibit mRNA translation. Nearly 750 of these small (18–25 nucleotides), non-coding RNA sequences have been identified in humans [3]. MicroRNAs are present in all systems, including the CNS, where they are involved in the regulation of nervous diseases and in neurotraumatic pathologies such as Alzheimer's, Parkinson's, and Huntington's diseases, Tourette's syndrome and schizophrenia [4]. Preliminary studies using microarray analyses to examine microRNA expression profiles post-SCI in mice [5] and rats [6] have confirmed significant and common changes in the expression of several microRNAs (*e.g*., miR-21 overexpression) and have identified potential downstream targets for some of these [6].

In this study, we performed a detailed analysis combining the use of microarrays, Q-PCR and several bioinformatic tools to characterize the microRNA expression changes induced by spinal cord contusion in a rat model. To deepen our understanding of the evolution of microRNA expression patterns following SCI, we examined microRNA expression at 3 different time points after injury (1, 3, and 7 days) and compared the expression levels at these times with those from untreated controls and surgical controls (shams). Our results reveal that a progressive microRNA underexpression occurs following SCI, and becomes particularly significant 7 days after injury.

This temporal pattern is closely correlated with the increases in mRNA expression observed 7 days after injury in previous studies [7], [8]. Moreover, a computational re-analysis of previous mRNA expression data predicted some of the observed microRNA expression changes, strongly suggesting a role for microRNA regulation in the processes that occur post-SCI. Integration and annotation of these data allow for the prediction of microRNA regulation in different pathways such as inflammation, nervous system development or cell death. Taken together, the data presented here strongly suggest that microRNAs are involved in different pathophysiological processes that are triggered after SCI, suggesting that the modulation of microRNA expression may be a promising therapeutic tool.

## Results

### Experimental spinal cord injury induces a motor medullary paralysis of medium grade

**Figure 1:**
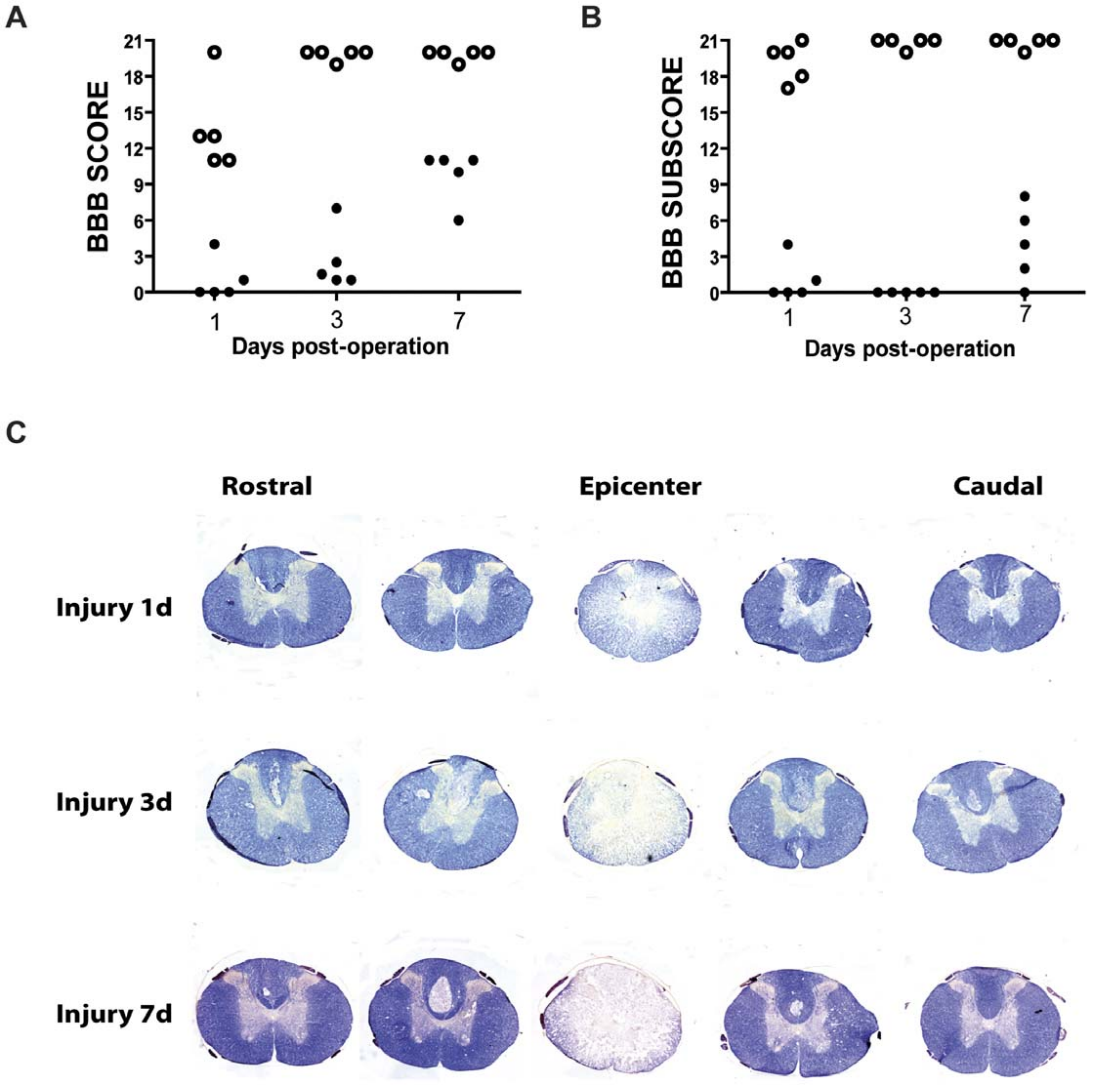
Functional and morphological outcomes after injury. Recovery of locomotor function as represented by BBB scores (A) and subscores (B) after moderate injury and sham surgical procedure. Open circles represent sham animals and closed circles represent injured animals. (C) Representative histological images of eriochrome/cyanine-stained spinal cords from the different experimental groups. Tissue samples show the site of impact and 0.5 cm rostral and caudal sections with a separation of 200 µm between each image.

Wistar rats received a moderate contusion (200 kdyne) at the level of the 8th thoracic vertebra (T8) to produce a reproducible spinal cord injury. Prior to sacrifice, injured rats were evaluated for hind-limb locomotion using the Basso, Beattie and Bresnahan locomotor rating scale (BBB scale) [9] and its subscore scale as modified by McTigue and collaborators (2007) [10]. Individual BBB scores and subscores were plotted as functions of the length of time after injury. We observed that all groups presented homogeneous values, and the means of the BBB scores agreed with the moderate spinal cord injury values obtained in previous studies [7], [9], [11] (Figure 1A, B). The injured animals presented some degree of spontaneous recovery of motor function as the time after injury progressed. An additional group of animals were used to assess histopathology, and we observed that the injury size of the area adjacent to the injury increased with the length of time post-injury (Fig. 1C).

### MicroRNA expression profiling of intact, sham, and injured rat spinal cords

**Figure 2:**
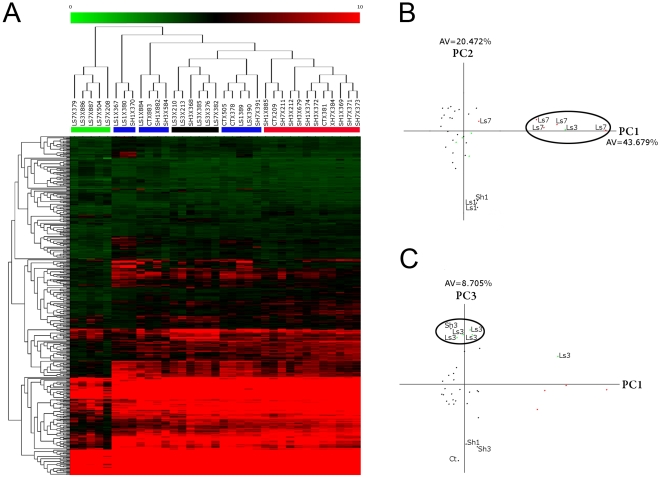
MicroRNA expression profiles after spinal cord injury. (A) Hierarchical cluster analysis and heat map, Euclidean distance and average linkage clustering of data from individual replicates. This analysis provides a visual ordination of the samples and genes according to their overall similarity. The colors of the heat map indicate microRNA upregulation (red) and downregulation (green). (B) PCA and scatterplot of the first two components, showing the separation of the LS7 individuals. (C) Scatterplot of the first and third components of the PCA, showing a group of LS3 samples. All analyses were based on data from the 463 microRNAs showing variable expression (IQR>0.5). PC1, PC2, and PC3 correspond to the axis determined by the first, second and third principal components of the PCA, respectively. These components are lineal combinations of the expression values for each gene optimized to capture the maximum variation of the matrix. Each consecutive component is orthonormal to the previous ones and absorbes the maximum amount of the remaining gene expression variation (AV, absorbed variation). Ls7, Ls3, Ls1, Sh1, Sh3 and Ct indicate sample type and correspond to Lesion (Ls), Sham (Sh) and control, while the numbers 1, 3, 7 indicate the sampling time after surgery.

MicroRNA expression data were obtained after RNA samples were hybridized to Exiqon miRCURY LNA microRNA v.11 microarrays carrying probes for 1,773 unique microRNA sequences. This microarray set comprises all human, mouse, and rat microRNAs that are included in the miRBase database v. 11.0 (http://www.mirbase.org), in addition to some viral sequences and novel human mature sequences identified by Exiqon (hsa-miRPlus probes). RNA from the spinal cord at the T8 level was extracted 1, 3, and 7 days after either laminectomy and contusion injury (lesion animals) or after laminectomy without contusion (sham animals). An additional group of animals that did not receive surgery was included as a representation of naive conditions (control animals). RNA was obtained from 5 animals in each of the 7 resulting groups. The processing and normalization of the hybridization data were performed using the variance stabilization normalization (VSN) methodology to obtain microRNA expression profiles for the 35 samples (Figure 2). The raw and the VSN-transformed data are available at the GEO database (http://www.ncbi.nlm.nih.gov/geo, last accessed in July 2011), under the GSE19890 accession code. On average, approximately 500 microRNAs were detected at levels above background in each sample, corresponding to an average of 28% of the microRNA probes included in the array. In total, microRNA expression was detected by 3,643 out of the 7,091 total microarray probes, corresponding to 901 microRNAs and representing roughly 51% of the microRNAs under investigation, including 268 rat sequences (77% of the 349 rat probes included in the array). Of the 901 detected microRNAs, 463 showed variable expression levels across samples, with an interquartile range above 0.5. An Excel file including all matrices and processing information is available as supplementary material (file S1).

Principal components analysis (PCA) and unsupervised, hierarchical clustering analysis (HCL) of the samples and the hybridization levels of the 463 microRNAs showing detectable and variable expression revealed a clear segregation pattern of the samples. Both PCA and HCL are multivariate methods commonly used to analyze the behaviour of multiple variables at the same time. They are routinely used in microarray analysis to explore the behaviour and grouping patterns of samples and genes according to the expression data. According to both analyses, samples obtained 7 days after spinal cord injury appeared as clearly separate from all other samples. As illustrated in Figure 2, both the first component of the PCA analysis and the branching pattern of the heat map revealed a group comprising 4 out of the 5 animals sampled at 7 days postoperation (LS7 individuals) as well as 1 LS3 individual (sampled at 3 days after injury) that are clearly separate from all other individuals. A group consisting of predominantly LS3 individuals could also be recognized in the heat map as well as by the third principal component of the PCA, whereas most untreated (control, CT) and sham individuals (SH) tended to group together. Samples collected from animals 1 day after injury (LS1) did not group together, either according to any PCA component or the heat map, and these LS1 samples could not be distinguished from those of the control or sham animals. The PCA results also revealed that the first dichotomy between the total samples and the LS7 samples is mostly due to the microRNA downregulation in the LS7 samples (see file S2). The latter samples only showed increased expression in a restricted number of microRNAs, particularly miR-21. The presence of one LS3 individual within the LS7 group and one LS7 individual out of this group, as well as the mixture of LS1 samples together with sham and control individuals, does not correlate with any biological (*i.e.*, BBB score) or processing (*i.e.*, RNA, hybridization, or image quality) variable evaluated, and we therefore assume that this response is due to biological variations not considered in this study.

### Spinal cord injury causes a progressive decrease in microRNA expression

**Figure 3:**
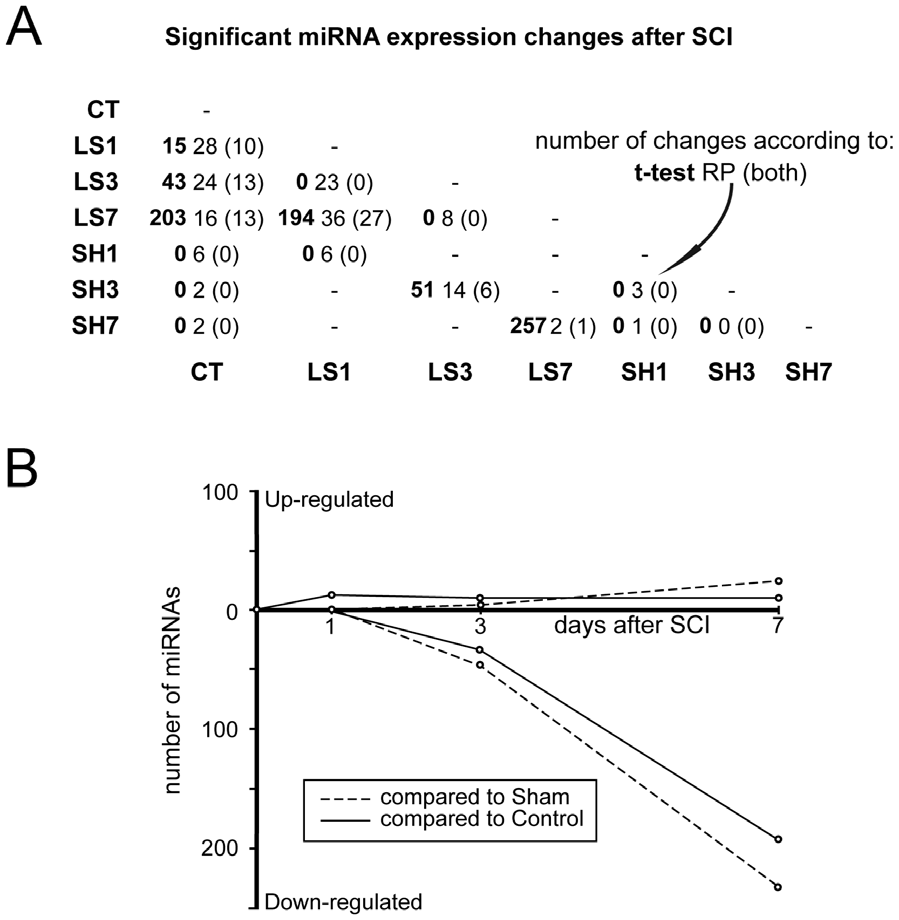
Number of microRNA expression changes following spinal cord injury. (A) Table showing the number of expression changes detected for the different pair comparisons performed. For each comparison, the first number (in bold type) corresponds to the number of significant changes according to t-test analyses after FDR adjustment; the second number (in regular type) corresponds to the number of significant changes according to the non-parametric Rank Product test; the third number (between brackets) corresponds to the changes found to be significant by both tests. (B) Scatterplot illustrating the changes in microRNA expression after SCI. The y-axis indicates the number of up-regulated (positive axis) and down-regulated (negative axis) microRNAs. Values were derived from the t-test analyses comparing microRNA expression levels at different times after SCI to the corresponding sham or control data.

Expression of individual microRNAs was compared between groups, and these results are presented in Figure 3. A paired Student t-test followed by False Discovery Range correction identified 763 significant changes in the studied comparisons, affecting 343 different microRNAs (a full list is provided in file S3). The distribution of these changes reveals that spinal cord injury induces progressive changes in microRNA expression patterns, which begin 3 days after injury and increase with time after surgery (Figure 3B). This pattern results from a gradual increase in the number of downregulated microRNAs, whereas the number of upregulated microRNAs remains low throughout the analyzed time period (Figure 3B). MicroRNA downregulation is particularly evident 7 days after injury, which is in agreement with the results from the PCA and HC analyses. At this 7-day time point, roughly 200 microRNAs are downregulated with respect to the control or sham levels and also compared to the LS1 expression levels, although there are no significant changes with respect to the LS3 levels. In contrast, the LS3 and LS1 animals revealed far less significant changes in microRNA expression levels with respect to those of the sham and control groups, and no significant differences were found between control and sham individuals or between sham individuals analyzed at different time points.

In addition to providing information regarding general expression patterns, the above comparisons also allowed us to identify individual microRNAs with changes in expression following SCI. To confirm the altered expression profiles of microRNAs identified with the t-test, we performed comparisons between the same pairs using the non-parametric Rank Product analysis [12]. These analyses detected fewer significant changes than did the t-test and identified a total of only 170 changes in 88 different microRNAs. Agreement between both tests (Figure 3A) was found for only 70 expression changes involving 53 different microRNAs. These 53 microRNAs include members of the miR-17-92 cluster, miR-21, or the nervous system-specific miR-124 (see the table in file S3).

### Changes in microRNA expression are reflected in mRNA profiles

**Figure 4:**
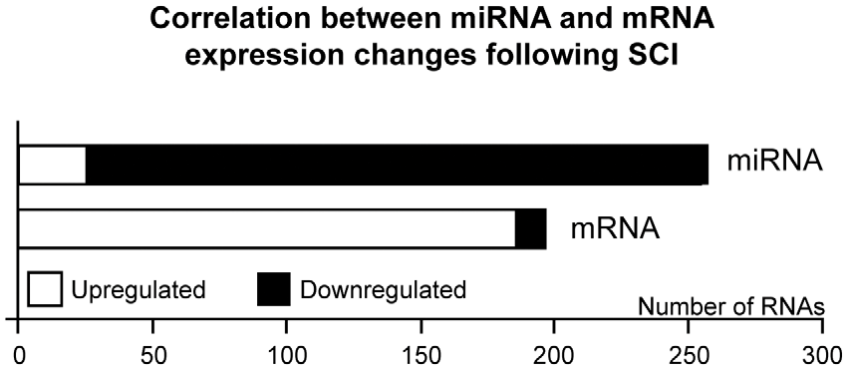
Negative correlation between microRNA and mRNA expression changes. Graph illustrating the relationship between the changes in microRNA and mRNA expression 7 days after injury. It clearly shows that mRNA upregulation parallels decreased microRNA expression. Numbers of upregulated and downregulated microRNA data according to the t-tests comparing the injured and sham animals at 7 days after SCI. The mRNA data correspond to the equivalent comparisons (mild plus moderately injured vs. sham at 7 dpo) from DeBiase *et al.* [7].

MicroRNAs mediate the post-transcriptional regulation of gene expression, and there is an inverse correlation between their expression and that of their mRNA targets [13], [14]. To confirm the effects that the observed microRNA expression changes had on the transcriptome, we assessed a list of microRNA targets, according to the miRanda algorithm (available at http://www.microrna.org/microrna/getDownloads.do) and the mRNA expression data from De Biase *et al*. [7]. These authors described spinal cord gene expression patterns at different times after mild, moderate and severe contusions. The injuries in the present study correspond to the mild and moderate lesions from the study by De Biase, according to the BBB scores. A general comparison between these microRNA and mRNA profiles showed that the microRNA downregulation following SCI in the present study is in parallel to the mRNA upregulation resulting from equivalent injuries (Figure 4).

Considering only the expression changes occurring 7 days after SCI in the present study, 59 out of the 187 upregulated genes following moderate and mild injuries are targets of microRNAs that demonstrate significant expression changes relative to the control at 7 days postoperation (dpo). A two-tailed Fisher exact test showed that this number of microRNA targets was significantly greater than would be expected by chance (see Table 1). This test also demonstrated that the targets of 25 microRNAs with changes in expression at 7 dpo from our analyses were also significantly enriched.

**Table 1:** Enrichment of microRNA targets 7 days after SCI.

	TNG	UP	%UP	Fp-val
**mRNA targets**	4315	59	1.367	<0.01
miR-103	359	7	1.950	<0.01
miR-107	359	7	1.950	<0.01
miR-10b	152	4	2.632	0.02
miR-128	331	8	2.417	<0.01
miR-133a	169	4	2.367	0.03
miR-138	308	9	2.922	<0.01
miR-145	299	6	2.007	0.02
miR-146a	268	5	1.866	0.04
miR-182	358	7	1.955	0.01
miR-185	522	8	1.533	0.03
miR-191	130	4	3.077	0.01
miR-219-2-3p	182	4	2.198	0.04
miR-300-5p	364	6	1.648	0.04
miR-325-3p	318	6	1.887	0.02
miR-329	200	5	2.500	0.01
miR-335	310	6	1.935	0.02
miR-340-5p	389	9	2.314	<0.01
miR-342-3p	229	5	2.183	0.02
miR-352	125	4	3.200	0.01
miR-376b-3p	214	6	2.804	<0.01
miR-382	246	6	2.439	<0.01
miR-494	316	8	2.532	<0.01
miR-7a	341	6	1.760	0.03
miR-96	237	5	2.110	0.02
miR-98	258	5	1.938	0.03
**Total mRNAs**	26379	187	0.709	

The table details the 25 microRNAs showing significant target enrichment among the mRNAs that were up-regulated 7 days after SCI according to De Biase *et al.* [7]. The p-values were calculated according to Fisher's Exact Test. TNG: total number of genes, UP: upregulated genes; %UP: percentage of upregulated genes; Fp-val: Fisher's exact test p-value.

To confirm this relationship, we used the methodology proposed by Cheng and Li [15] to infer microRNA expression changes from the mRNA expression data reported by De Biase *et al.* [7] to compare these results to our data. Using the data from these authors, the mRNAs showing significant changes in expression after mild and moderate lesions were used to predict the microRNAs potentially regulating these genes. According to these analyses, up to 21 microRNAs showed altered expression 24 hours after injury, and 14 were changed at 7 dpo (Table 2). A comparison between these identified microRNAs and those showing expression changes in the present microarray analysis revealed similar expression changes for miR-21 24 hours after SCI as well as for 4 other microRNAs, namely, miR-184, miR-340-5p, miR-369-3p and miR-466b, at 7 dpo (Table 2).

**Table 2:** MicroRNAs with expression changes inferred from the mRNA data.

miRNA	p-val	P.A.	DPO
rno-miR-23a	0	no	1
rno-miR-23b	0	no	1
rno-miR-338	0.004	no	1
rno-miR-219-2-3p	0.014	no	1
rno-miR-494	0.015	no	1
rno-miR-181d	0.018	no	1
rno-miR-433	0.022	no	1
rno-miR-210	0.025	no	1
rno-miR-743b	0.025	no	1
rno-miR-138	0.026	no	1
rno-miR-181b	0.027	no	1
rno-miR-130a	0.027	no	1
**rno-miR-21**	**0.03**	**yes**	1
rno-miR-328	0.034	no	1
rno-miR-664	0.035	no	1
rno-miR-181a	0.038	no	1
rno-miR-132	0.04	no	1
rno-miR-342-3p	0.043	no	1
rno-miR-466c	0.046	no	1
rno-miR-325-3p	0.047	no	1
rno-miR-154	0.049	no	1
**rno-miR-369-3p**	**0**	**yes**	7
**rno-miR-466b**	**0.01**	**yes**	7
rno-miR-320	0.015	no	7
rno-miR-543	0.018	no	7
rno-miR-152	0.022	no	7
rno-miR-674-5p	0.023	no	7
rno-miR-500	0.027	no	7
**rno-miR-340-5p**	**0.037**	**yes**	7
rno-miR-210	0.039	no	7
rno-miR-19b	0.039	no	7
**rno-miR-184**	**0.039**	**yes**	7
rno-miR-130b	0.04	no	7
rno-miR-221	0.045	no	7
rno-miR-872	0.046	no	7

List of microRNAs with expression changes predicted from the mRNA data from De Biase *et al.* [7]. All listed changes are associated with significant p-values according to the methodology of Cheng and Li [15]. Predicted changes were compared with the actual changes observed in the present analyses, as indicated in the P.A. (present analysis) column. “Yes” corresponds to changes observed in the present analysis, and “no” refers to changes that were not identified. DPO: days post-operation.

### Is microRNA expression tissue- or species-specific?

MicroRNA expression is known to be tissue-specific, and it may also be species- and strain-specific [16]. Phylogenetic variations in microRNA expression and function may thus limit their potential therapeutic applications. To evaluate potential phylogenetic inconsistencies in expression, we compared our results to published data from analyses describing microRNA expression profiles in the spinal cords of different species [6], [17], [18], [19], [20], [21], [22]. These comparisons reveal that most of the microRNAs with the highest hybridization values in the spinal cord from our analyses were also found in the spinal cords or central nervous systems of rats and other vertebrates in previous studies (see file S4). We observed major agreement between the present results and the data from Liu *et al.* [6] for Sprague-Dawley rats; of the 35 microRNAs with the highest expression levels in control animals, 22 (63%) were among the 50 microRNAs with the highest expression levels from our analyses, and the other 13 (37%) were also detected but were present at lower levels. Agreement was also observed when our data were compared to those from other vertebrate species, although the number of coincidences decreased. In these cases, approximately 20% of the microRNAs detected in the spinal cords of each of these species were not detected in the present study. These exceptions are shown in Table 3 and include, among others, the β catenin-related miR-200a and the cell-cycle regulator miR-663.

**Table 3:** MicroRNAs detected at the spinal cord of different vertebrates in previous studies [6], [17], [18], [19], [20], [21], [22] but not in the present study.

miRNA	rat	mouse	chicken	medaka	zebrafish
**miR-108**				X	X
**miR-128b**		X			
**miR-129**		X			
**miR-132**		X	X		X
**miR-137**					X
**miR-187**			X	X	
**miR-190b**				X	X
**miR-194**			X		
**miR-196a**					X
**miR-200a**			X		
**miR-202**		X			
**miR-205**			X		
**miR-206**			X		
**miR-210**				X	X
**mir-213**				X	X
**miR-216**					X
**miR-216b**				X	X
**miR-217**	X			X	X
**miR-221**			X	X	X
**miR-296**		X			
**miR-298**		X			
**miR-31**				X	X
**miR-324-3p**		X			
**miR-324-5p**		X			
**miR-326**		X			
**miR-328**		X			
**miR-346**		X			
**miR-351**		X			
**miR-370**		X			
miR-373*		X			
**miR-409-3p**		X			
**miR-423**		X			
**miR-430**				X	X
**miR-431**		X		X	X
**miR-433-5p**		X			
**miR-452**		X			
**miR-454a**					X
**miR-454b**				X	X
**miR-484**		X			
**miR-485-5p**		X			
**miR-500**		X			
**miR-638**		X			
**miR-663**		X			
**mir-7b**					X
**miR-92**		X	X	X	X

Note that no data from Liu *et al.* [6] are included in the table because some disagreements were observed among microRNAs with low expression but not among those with high expression (see supplementary table 1 from Liu *et al.*).

In contrast, microRNAs that were detected in the present study but not in previous studies (Table 4) include the Exiqon miR-plus probes as well as several newly released microRNAs, for which no previous data have been published, and more interestingly, the miR-451 cluster and miR-144, which are both important gene regulators of erythrocyte homeostasis and cardiomyocyte ischemia [23], [24].

**Table 4:** Spinal cord microRNAs exclusively detected in the present study, in either control (un-operated) or sham animals.

miRNA	CT	SH1	SH3	SH7
**miR-1280**	X	X	X	X
**miR-1308**	X	X	X	X
**miR-144**				X
**miR-1826**	X	X	X	X
**miR-1827**	X	X	X	X
miR-467e*	X			
**miR-665**		X		
**miR-923**	X			
**miRPlus-E1013**	X	X	X	X
**miRPlus-E1024**	X	X	X	X
**miRPlus-E1078**	X	X	X	X
**miRPlus-E1100**	X	X	X	X
**miRPlus-E1103**	X	X	X	X
**miRPlus-E1117**	X	X		
**miRPlus-E1218**	X	X	X	X
**miRPlus-E1252**	X	X	X	X
**miRPlus-E1253**	X	X	X	X
**miRPlus-E1258**	X			
**miRPlus-E1290**	X	X	X	X
**miRPlus-F1003**	X	X	X	X

Expression changes were less similar between studies, even within the same species and at the same time after injury. A comparison between the most significant changes detected by Liu and colleagues [6] and the present data (Table 5) shows that may microRNAs found to have altered expression in Liu's study were not detected here (only 50% of the microRNAs altered at 1 dpo and 35% of those at 7 dpo). For the remaining comparisons, most of the observed changes identified by the current study agree with those from Liu *et al.* [6] (25% at 1 dpo and 35% at 7 dpo). Some microRNAs showed opposite trends as miR-290, which was shown to significantly increase at 7 dpo, according to Liu's [6] analyses, whereas miR-290 showed a significant decrease in the present study. The remaining microRNAs showed non-significant changes in the present study, although most of followed trends were similar to those observed by Liu and colleagues [6].

**Table 5:** Comparison between the microRNA expression changes described by Liu *et al.* [6] and those detected in the present study.

Expression changes respect to control/sham				
	1 dpo		7 dpo	
Name	Liu	Present	Liu	Present
rno-miR-130b			1.42	NE
rno-miR-146a			1.72	**INC S**
rno-miR-15b			1.15	DEC NS
rno-miR-17			1.74	INC NS
rno-miR-18a	2.71	NE	3.41	NE
rno-miR-200c			4.12	NE
rno-miR-206			3.26	NE
rno-miR-20a			1.69	NC
rno-miR-20b-5p			1.83	NE
rno-miR-21			1.37	**INC S**
rno-miR-214			2.01	INC NS
rno-miR-219-5p			−1.82	**DEC S**
rno-miR-221			1.1	NE
rno-miR-223	3.58	**INC S**	3.4	**INC S**
rno-miR-24-2*			2.41	DEC NS
rno-miR-290	3.66	INC NS	2.96	**DEC S**
rno-miR-378			1.31	INC NS
rno-miR-410			−1.21	NE
rno-miR-466b			3.05	**DEC S**
rno-miR-541			1.11	**INC S**
rno-miR-874	2,8	NE		

Data restricted to microRNAs with significant changes in expression (2-fold or greater) according to Liu *et al*. [6]. Expression levels at 1 and 7 dpo are compared to the expression levels from control and sham individuals in the present study and to the sham individuals in the Liu study. NE, not expressed; INC, increase; S, significant; NS, not significant; NC, no change. Bold type denotes agreement in significant changes between Liu's and the present study.

### MicroRNA profiling data are consistent and reproducible

We validated the changes in the levels of the microRNAs miR-21, miR-223, miR-146a, miR-219-5p, miR-29c, miR-468, miR-145 and miR-107 using Q-PCR. MicroRNAs miR-21, miR-223, miR-146a, and miR-219-5p showed significant expression changes in our study (identified with both a t-test and a Rank Product test) as well as in other reports [6], [25]. The microRNA miR-21, which was the most highly overexpressed microRNA at 7 dpo, has been shown to play a role in apoptosis [26], [27], [28]. Similarly, we analyzed proapoptotic miR-29c, which regulates p53-mediated apoptosis [29], as well as miR-145 and miR-107, which belong to the group of 25 microRNAs whose targets were significantly enriched in the mRNA expression patterns following spinal cord injury reported by De Biase *et al.* (2005). miR-468 expression was not significantly altered in the arrays and is included here as a methodological control. Among the microRNAs listed above, the miR-219-5p level was within the most reduced microRNA levels at 7 dpo from the array data. Furthermore, the microRNAs miR-21 and miR-223 have previously been reported to be overexpressed in other nervous system array studies [5], [25].

**Figure 5:**
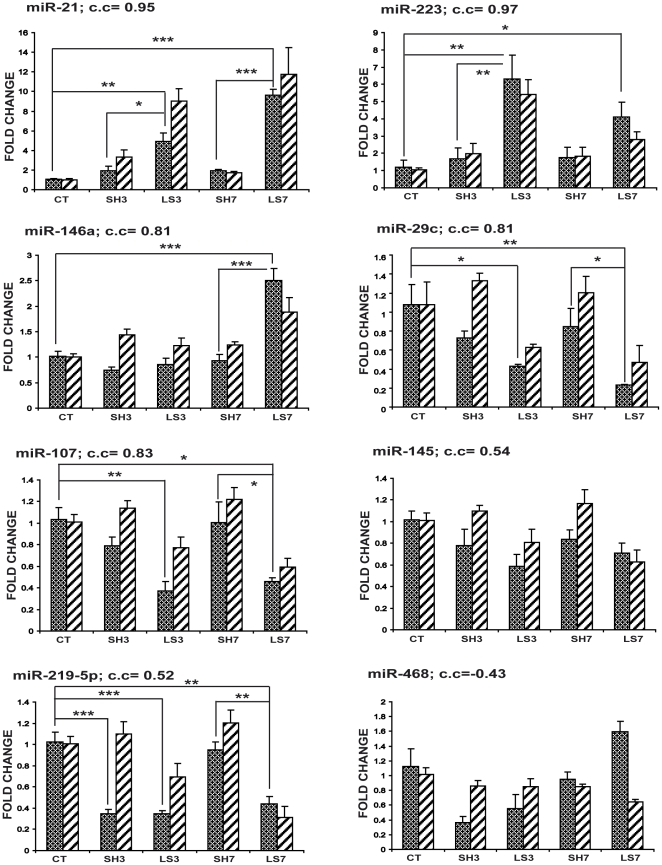
Temporal expression profiles of selected microRNAs. The expression levels of selected microRNAs were assessed using quantitative PCR (left columns) and compared with the corresponding microarray data (right columns) using a correlation analysis (c.c: correlation coefficient). The fold changes in the sham and injured experimental groups are with respect to the control group. The Q-PCR data were analyzed using a one-way ANOVA followed by a Tukey post-test. * = p<0.05; ** = p<0.01; *** = p<0.001.

We validated the expression of these microRNAs using TaqMan Real-Time PCR at 3 and 7 dpo in injured and sham animals, and then we compared both of these expression profiles to those from control animals (no surgery prior to RNA isolation). We used the small RNA U87 to normalize the total RNA expression. Figure 5 presents the correlation between the microRNA expression patterns obtained from the array and the normalized Q-PCR data.

The Q-PCR analysis from animals sacrificed at 3 days postoperation revealed that miR-21 and miR-223 are significantly upregulated in injured animals compared to both control and sham animals. For the microRNAs that were downregulated at 3 dpo, miR-29c and miR-107 were significantly repressed in the injured animals compared only to the control group, whereas miR-219-5p was significantly downregulated in comparison to both the control and the sham groups. For miR-146a and miR-145, no significant variations in expression were found, although there was a trend toward downregulation. When the changes in expression were analyzed at 7 dpo, miR-21 and miR-146a levels were found to be significantly increased at 7 dpo in comparison to the control and sham groups. Similarly, miR-223 levels significantly increased at 7 dpo compared to those of the control group but not compared to those of the sham group. Conversely, the expression of miR-219-5p, miR-107 and miR-29c were repressed at 7 dpo. For miR-219-5p, miR-107 and miR-29c, there was a strong repression in injured animals that was significant when compared to both the control and the sham animals. The expression level of miR-145 also showed a repression trend, although this was not significant (Figure 5).

### MicroRNAs affect several functions and pathways after spinal cord injury

Spinal cord injury affects different biological processes and pathways at different times after injury via changes in gene expression. To evaluate the influence of microRNA level on these gene expression changes, we employed two approaches based on the Gene Set Enrichment Analysis (GSEA). The first approach follows that of Gusev [30], [31] and refers to the targets of microRNAs that demonstrate significant changes in expression regarding the inferred Gene Ontology (GO) terms for biological processes. This method allows for the identification of GO terms that are significantly altered according to the observed microRNA expression differences between the control, sham and injured animals. The complete list of GO terms that were significantly enriched for each comparison and the number of microRNAs that were involved are included in file S5. The top 10 most significantly enriched terms for all comparisons included those related to homeostasis, responses to endogenous stimuli, transcription and the transmission of nerve impulses (see Table 6).

**Table 6:** Biological effects of the microRNA expression changes.

	comparisons					
GO term	C1	C2	C3	C4	C5	C6
GO:0051272∼positive regulation of cell motion	x					
GO:0010033∼response to organic substance	x	x	x	x	x	
GO:0030335∼positive regulation of cell migration	x					
GO:0015031∼protein transport	x				x	x
GO:0045184∼establishment of protein localization	x					x
GO:0016337∼cell-cell adhesion	x					
GO:0040017∼positive regulation of locomotion	x					
GO:0007156∼homophilic cell adhesion	x					
GO:0051270∼regulation of cell motion	x					
GO:0048878∼chemical homeostasis	x					x
GO:0042981∼regulation of apoptosis		x				
GO:0006350∼transcription		x	x	x	x	x
GO:0015672∼monovalent inorganic cation transport		x				
GO:0008104∼protein localization		x				x
GO:0050670∼regulation of lymphocyte proliferation		x	x			
GO:0045449∼regulation of transcription		x				
GO:0009719∼response to endogenous stimulus		x	x	x	x	x
GO:0007242∼intracellular signaling cascade		x		x	x	
GO:0031644∼regulation of neurological system process			x			x
GO:0051970∼negative regulation of transmission of nerve impulse			x			
GO:0044057∼regulation of system process			x			
GO:0050805∼negative regulation of synaptic transmission			x			
GO:0010648∼negative regulation of cell communication			x			
GO:0042698∼ovulation cycle				x		
GO:0019228∼regulation of action potential in neuron				x		
GO:0009725∼response to hormone stimulus				x	x	
GO:0007267∼cell-cell signaling				x		
GO:0050767∼regulation of neurogenesis				x	x	
GO:0042592∼homeostatic process					x	x
GO:0019226∼transmission of nerve impulse					x	x

List of the 10 Gene Ontology biological function terms that were most significantly enriched according to the targets of microRNAs with significant expression changes, according to the t-test analyses. The GSEA values were obtained using DAVID (http://david.abcc.ncifcrf.gov) algorithms. Comparisons are as follow: **C1**) Comparison between CT and LS1; **C2**) CT vs LS3; **C3**) SH3 vs LS3; **C4**) CT vs LS7; **C5**) SH7 vs LS7; and **C6**) LS1 vs LS7.

**Figure 6:**
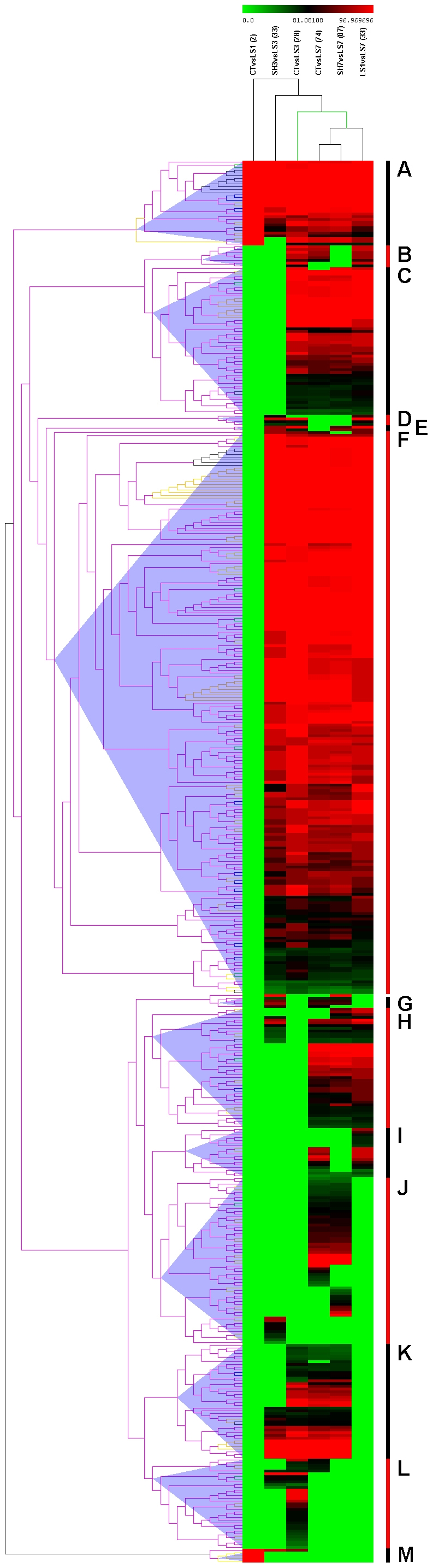
Functional involvement of microRNAs with expression changes. A heat map of unsupervised hierarchical clustering of all Gene Ontology (GO) categories obtained from an enrichment analysis of the microRNAs with significant changes among groups according to the t-test analyses. The GSEA values were obtained using DAVID (http://david.abcc.ncifcrf.gov) algorithms. The gradient represents the percentage of microRNAs targeting each category in each comparison. The GO terms included in each cluster (A to M) are detailed in file S5.

To further explore these data, we followed the method of Gusev *et al.* [31] and performed a hierarchical clustering using the comparisons as the samples and the numbers of microRNAs in each significantly enriched GO term as the variables (the data matrix is provided in file S5). The resulting heat map (Figure 6) shows a pattern with distinct, well-defined groups. The graph reveals a group located within the uppermost region (labeled group A), which included all GO terms that were altered in response to injury (with respect to the sham or controls), irrespective of the time point considered, as well as those altered between 1 and 7 days after injury. These 31 GO terms represent biological processes related to cell migration, neurotransmitter transport, transcription, responses to various stimuli and terms related to cell growth and adhesion. A second group (group M), which was located at the bottom of the heat map, included biological processes that were exclusively altered at 1 day after injury. These 4 GO terms are related to cell adhesion, muscle cell migration and the protein kinase B signaling cascade. Conversely, there was a large group of GO terms representing biological processes (group F, 204 GO terms) that were altered at 3 or more days after injury. This group includes GO terms related to responses to various extracellular stimuli, the regulation of cell death and apoptosis, negative regulation of nerve impulses, neuron differentiation, ion homeostasis as well as other aspects of the cell growth including cell size, proliferation, cell cycle or morphogenesis. Group C consisted of 54 GO terms that were altered as a result of the same comparisons as in group F but excluded terms corresponding to the comparison between LS3 and SH3. These GO terms that were altered in response to middle-to-late injury represented biological processes related to myelinization, neurogenesis, neuron differentiation, neuron projections, and vascularization, together with more general processes such as cell motion, cell growth and development. Group H includes GO terms that were altered in all comparisons involving LS7 individuals (*i.e*., LS7 individuals compared to the control, SH7 or LS1 individuals). The most remarkable terms represent neuron projection development, neuron migration, synaptic transmission and plasticity, and pain perception in addition to more general terms involving stimulus responses, morphogenesis and the regulation of cell differentiation. Group J includes GO terms that were altered 7 days after injury relative to the controls and shams (either at the same time or for each comparison) as well as the terms that were unique to the comparison between LS3 and SH3. These 61 GO terms are fairly diverse and are related to general biological processes, such as regeneration and cell division, as well as calcium ion and stress responses, immune responses and many others with little or no relation to nervous tissue. Another interesting group, labeled group K, includes terms that were altered based on the comparisons between the LS3 and LS7 individuals relative to the CT and sham individuals. This group includes GO terms related to glial cell genesis and differentiation, wound healing, regeneration, and the responses to various stimuli including cAMP.

Similarly, we used IPA 5.0 (Ingenuity Systems, Redwood, CA) to predict molecular networks involving the altered microRNAs, their targets, and their related functions. The IPA analysis identified gene interaction networks related to biological processes (*e.g*., programmed cell death, organism injury, and transcriptional activation) that were associated with the differentially expressed microRNAs between the control and experimental groups. Detailed information for the resulting networks obtained using the IPA enrichment analysis is provided in file S6.

**Figure 7:**
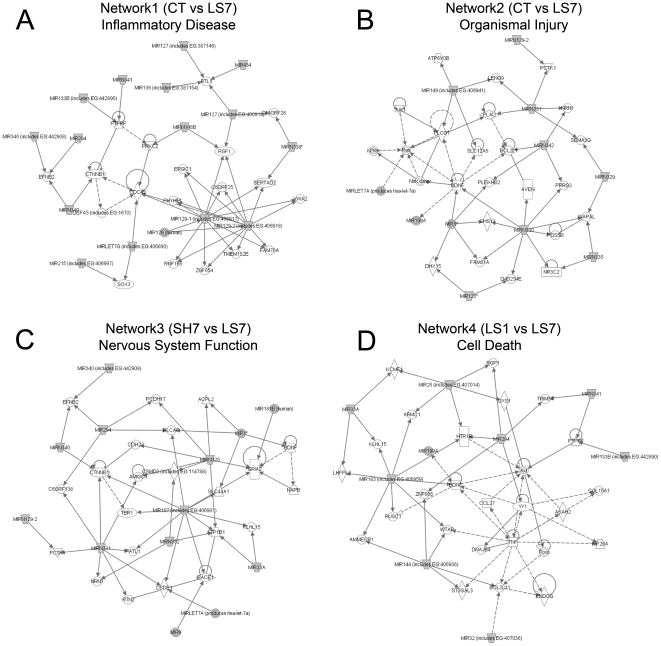
Selected networks affected by microRNA expression changes according to Ingenuity Pathway Analysis (IPA). Each node represents molecules (microRNAs, gene) and the biological relationship between two nodes is represented as a line. These networks include microRNAs with expression changes obtained by comparison of the injury 7dpo group (LS7) with respectively non-injured control group (CT)(network 1 and 2), Sham 7dpo group (SH7)(network3) and injury 1dpo (LS1) (network4). The top four functions associated with networks are (A) Inflammatory Disease, Cell Morphology, Cellular Assembly and Organization, (B) Organismal Injury and Abnormalities, Cardiac Damage, Cardiovascular Disease, (C) Nervous System Development and Function, Tissue Morphology, Cellular Development and (D) Cell Death, Nervous System Development and Function, Cellular Function and Maintenance.

Following the IPA analysis, we observed that molecular networks with top-ranking IPA scores were mainly those involving the LS7 and LS3 groups. Interestingly, these results indicate that many top-ranked biological functions and diseases were associated with major post-injury events triggered by SCI. Hence, many networks related to cell death, inflammation, organism injury and nervous system development and function were generated, and the most relevant networks are depicted in Figure 7.

We observed downregulation of microRNAs related to cell cycle genes such as *cdc42* (miR-LET7g) and *ctnnb1* (miR340, miR-331), which is consistent with many cell cycle-related genes that have been implicated in neuronal damage after spinal cord injury [8]. We also found that other genes related to inflammation or cell death within the same pathways were also targeted by microRNAs. For example, the regulation of NF-κB activation *in vivo* by PRKCZ (network 1) [32] is related to the downregulated miR-148b, and the downregulation of miR-133b and miR-341 is related to PTPRF (Figure 7, networks 1 and 4), the overexpression of which induces apoptosis independently of p53 through a caspase cascade that does not affect cell adhesion [33]. Similarly, several downregulated microRNAs were associated with genes participating in cell death process, such as *yy1* (miR-294) [34], the pro-apoptotic factor *bim* (miR-144), the pro-apoptotic *blcap* (miR-294), which stimulates apoptosis independently of p53 and NF-κB [35], and the anti-apoptotic factor Bcl-XL (miR-342) (Figure 7, networks 1, 3 and 4). It is interesting to note that some microRNAs found in the IPA networks have been implicated in the regulation of inflammation and cell death pathways. Specifically, miR-Let7a, miR-107, and miR-183 have been described as regulators of cell death, and miR-181b and miR199 are involved in the control of inflammation (see file S6).

Levels of chondroitin sulfate proteoglycans, which are neurite growth inhibitors present in scars surrounding injury, increased after SCI [36]. This increase functions to limit the regenerative process of the spinal cord. Interestingly, in network 2, we detected signaling components involved in extracellular matrix organization, such as PLAU (miR-331) and CHSY1 (miR-1*, miR-330).

In addition to these findings, the expression of growth factors as well as neuritogenic and axonal guidance molecules has also been described in the spinal cord following denervation or nerve injury [1]. According to the IPA networks (see Figure 7), the gene expression of many of these factors is related to different microRNAs, which are downregulated following SCI according to our analyses. Specifically, BDNF is related to miR-183, semaphorin 3 to miR-329 and miR-331, Neuritin-1/*nrn1* to miR-331 and miR-342 and Ephrin-B2/*efnb2* to miR-340 and miR-294. The expression of these molecules involved in axonal targeting, neuronal survival and neurite outgrowth reflects the response of the spinal cord to damage and the initiation of repair.

## Discussion

MicroRNAs are small non-coding RNA molecules (18–24 nucleotides long) that regulate gene expression by interacting with specific sequences of target mRNA or promoters [13], [14]. These post-transcriptional regulators can be tissue- and cell–specific and serve to establish and maintain the characteristic protein expression profiles of distinct cellular phenotypes [37]. According to some studies, the CNS expresses more distinct microRNAs than any other tissue [18], [38], in accordance with the roles of these molecules in CNS development and neural function (see [39], [40] and references therein). MicroRNA dysregulation in the CNS is associated with neurodegenerative disorders, including Alzheimer's, Parkinson's, and Huntington's disease, as well as traumatic injury [37], [41], [42].

In this report, we describe the effects of moderate spinal cord injury on microRNA expression at the injury site 1, 3 and 7 days after the insult. Our analyses show that moderate SCI causes an extended microRNA dysregulation, which affects multiple processes that are in many cases related to secondary damage from SCI. The observed expression changes consist mainly of increasing numbers of down-regulated microRNAs, whereas only a few microRNAs appear to be upregulated. This pattern becomes apparent 3 days after injury and is evident at 7 dpo, whereas few changes are detected 1 day after injury. Progressive microRNA dysregulations have been previously described in contusive spinal cord injuries [6] and in the rat hippocampus following ischemia [43]. Moreover, Strickland and colleagues [44] observed a similar increase in the number of downregulated microRNAs up to 14 days after spinal cord injury.

Identifying the causes underlying the SCI-induced microRNA downregulation is complex due to the heterogeneous cell composition of the spinal cord and the multiple changes that take place after injury. Expression changes in heterogeneous tissues may be ascribed either to changes in gene regulation within a given cell type or to changes in the relative abundance of the expressing cell types [45]. The observed microRNA underexpression cannot be directly linked to tissue loss because an equivalent amount of RNA is hybridized to the arrays to create comparable global microRNA hybridization signals (estimated as the mean expression value from all microRNA probes on each array; data not shown). After trauma, the spinal cord may experience changes in the relative proportions of different cell types due to the necrotic and apoptotic death of neurons and oligodendrocytes [46], [47] or to the infiltration of immune cells [2]. The death of specific cell types may explain the downregulation of microRNAs that are associated with neurons, such as miR-124 and miR-128 [48], and those associated with oligodendrocytes, such as miR-219, miR-138, and miR-338 [49], [50]. In parallel, the infiltration of immune cells, such as neutrophils and macrophages, may also explain the overexpression of miR-223 after spinal cord injury [25]. Thus, changes in the proportions of cell types present in the spinal cord can alter the microRNA expression pattern. However, this contribution is probably minor, as a major contribution would result in a generalized shift in microRNA expression rather than an extended downregulation of microRNA expression, as was observed in our study. In fact, according to previous studies [17], [18], [19], [21], [22], highly expressed microRNAs in the spinal cord or the CNS, such as miR-125b, miR-29a, miR-30b, and miR-9*, show sustained, high levels of expression before and after injury (see file S1), suggesting an overall preservation of the cell populations in the spinal cord. Additionally, changes in microRNA expression in these cells may also contribute to the observed profiles. Global microRNA downregulation as a cellular response has been observed in cancers [51], [52], [53], in stimulated effector T cells [54], and in CNS neurons after exposure to noxious stimuli, both *in vivo* and *in vitro* [55]. Moreover, the observed correlation between low microRNA levels and high mRNA levels following spinal cord injury (see Figure 4) strongly supports microRNA regulation of mRNA levels, although we cannot rule out the effects of changes in the proportions of different cell types. Cells can also reduce microRNA abundance by blocking the required maturation machinery, e.g. inhibiting DICER or DROSHA expression. Recent evidence has shown that cancer cells [53] downregulate their microRNA network by targeting DICER through the overexpression of the microRNAs miR-103 and miR-107. However, this mechanism likely does not apply to the present case because miR-103 and miR-107 appear to be downregulated following injury, and previous studies have not found significant changes in DICER or DROSHA expression [7].

Reduction in microRNA abundance appears to be a general characteristic of cancer [51], [52], [53], [56], and it is also observed in response to noxious agents [57] and in some neuropathies [58]. MicroRNA downregulation in cancer cells induces tissue plasticity and fosters invasive and metastatic behaviors [53]. Similarly, global microRNA downregulation induced by DICER ablation precludes the differentiation of neural stem cells [59], [60] and the acquisition of myelinating phenotypes in oligodendrocytes [49]. In adult neural cells, induced microRNA reduction causes the death of mature neurons [61] and oligodendrocytes [62], alters the transcriptome of astrocytes such that they resemble more immature or reactive-like states [63], and can even lead to changes in neural plasticity associated with memory and learning ability [64]. Thus, global microRNA downregulation may underlie common processes observed after spinal cord injury, such as neural and oligodendrocyte cell death and astrocyte reactive gliosis, and may even be involved in neuronal plasticity.

We explored the functional roles of the microRNAs that are dysregulated after SCI using different bioinformatic approaches [30], [31], [65] based on the identification of Gene Ontology terms and signaling pathways potentially regulated by co-expressed microRNAs. These analyses revealed that changes in microRNA expression affect a large group of biological functions known to be altered following SCI [2], [47], including alterations in general processes such as transcription, cell growth, and migration. Specific analyses of microRNA expression that demonstrated altered expression at 3 or more days after injury indicate that these microRNAs may regulate key processes including cell death or apoptosis, nerve impulses, the cell cycle, wound healing, ion homeostasis, responses to external stimuli (including the immune response), myelinization, neural cell genesis and differentiation, and vascularization.

Among these functions, cell death due to apoptosis or other pathways is a hallmark of the pathophysiology of SCI [66], [67]. Unlike the necrotic cell death that accompanies the primary damage, apoptotic cell death is a gene-controlled event that is stimulated or inhibited by a variety of regulatory factors including several microRNAs [68]. Accordingly, our analyses suggest that changes in the expression of approximately 20 microRNAs at 3 and 7 days after spinal cord injury are involved in the regulation of cell death through different pathways, including those networks identified by the IPA analysis (Figure 7). A close inspection of the known effects of these microRNAs reveals a complex situation, involving expression changes that could potentially simultaneously stimulate and inhibit apoptosis (see Table 7). Apoptosis may be stimulated by the downregulation of up to 7 protective microRNAs as well as the upregulation of the pro-apoptotic miR-15b microRNA at 3 days after injury. Conversely, we would expect apoptosis to be inhibited by the downregulation of many pro-apoptotic microRNAs together with the upregulation of members of the miR-17-92 cluster. The effect of changes in the expression of other microRNAs, such as members of the let7/miR-98 family, remains controversial because they display variable roles in apoptosis depending on the circumstances (see Table 7). According to previously published studies, most of these microRNAs regulate apoptosis through the p53 or AKT pathways or by silencing key apoptosis molecules, such as caspases 3 and 9, Fas/CD95, c-Myc, or several members of the BCL2 family of proteins. The roles of these microRNAs in secondary cell death should be accompanied by coherent changes in the expression of their apoptosis-modulating targets in the spinal cord. This would likely be the case for the observed increases in the expression of the cell death inducers caspase 3 [69], [70] and Fas [71], the expression of which parallels the decreased expression of their regulators, the let7/miR-98 family members miR-96 and miR-146a. Similarly, HSP70 has been shown to demonstrate a strong increase in expression peaking at 7 days after injury [69], which directly matches the reduced expression of its regulator miR-1 [72]. Additionally, the overexpression of the mitochondrial superoxide dismutase 2 gene (*sod2*) 7 days after injury [73], [74] is consistent with the downregulation of its modulator miR-145 [75]. More interestingly, the downregulation of the BCL-2-inhibiting microRNAs miR-1, miR-138, and miR-148b is broadly consistent with the increase in the number of BCL-2-positive cells present 3 days after injury ([76]; however, see [77]), although microRNA downregulation extends throughout the 7-day period after injury, which is typically a time when the number of BCL-2-positive cells is progressively reduced. Regulation of BCL-2 by miR-107 and other microRNAs was recently discussed in a profiling study by Liu *et al*. [6]. However, contrary to the current evidence, these authors observed a miR-107 upregulation 4 hours after injury, which they proposed should decrease BCL-2 levels and induce apoptosis. Disagreements extend to other cell death-related microRNAs that were reported by Liu [6] to demonstrate changes in expression that we were not able to detect (mir-137 and miR-672) or whose expression did not seem to change in the present study (miR-214, miR-30-3p, miR-235-3p, and miR-674-5p). The causes underlying these discrepancies with previous microRNA profiling studies may be related to strain differences or, more likely, to differences in the severity of the injury, as discussed below.

**Table 7:** Apoptosis-related microRNAs with significant changes in expression in the present study.

MicroRNA	Expression Changes	Targets, effects and references
*Pro-apoptotic*		
miR-1	D3, D7	BCL-2 [117], HSP60 and HSP70 [72], IGF-1 [118], [119]
miR-7	D3	EGFR regulating AKT pathway [120]
miR-15b	U3	BCL-2 [121], [122]
miR-29b	D3	MCL-1 [123], [124], activate P53 pathway [29]
miR-34	D3, D7	SIRT1 [125], BCL-2 [126],
miR-101	D7	MCL-1 [127]
miR-103/107	D7	CDK5R1 [128]
miR-138	D3, D7	BCL-2 [129]
miR-145	D7	c-Myc [130], see also [131] and [132]
miR-183	D7	PDCD4 inhibit TGF-beta-induced apoptosis by downregulation of PDCD4 [133]
miR-184	D7	AKT2 [109]
miR-204	D7	BCL-W, BIRC2 [134]
*Anti-apoptotic*		
miR-10	D7	BIM [135], [136]
miR-17-92 cluster (miR-17 & 20)	U3	BIM, E2F1 protective according to [139], [140]
miR-21	U3, U7	TPM1, PTEN [148], PDCD4 [98], pro-apoptotic [149]
miR-96	D7	FOXO1 [144], CASP-3, FADD [27]
miR-125b	D7	BMF [137], ERBB2, ERBB3 [138]
miR-146a	U7	FAS [141], promotes survival [142]
miR-191	D7	Inhibition causes death of cancer cells [143]
*Both pro-apoptotic and anti-apoptotic*		
Let7/miR-98 family	D7	CASP-3 [145], FAS [91], BCLXL [146], c-Myc [147]
miR-133b	D3, D7	CASP-9 [72], BCL-W, MCL-1 [150]

Expression changes, targets and references for the pro-apoptotic and anti-apoptotic microRNAs and those exhibiting dual roles are detailed. For each microRNA, the expression changes in the present study are detailed in column EC (expression changes; D corresponds to downregulation and U to upregulation at the indicated dpo).

Recent studies have provided compelling evidence that microRNAs are involved in the immune response [78], [79]. SCI triggers an inflammatory response that is initiated by an alteration of the blood-brain barrier and followed by the sequential infiltration of various peripheral immune cells, the activation of microglia and the subsequent induction of inflammatory pathways. According to our functional analyses, expression changes in several microRNAs may participate in these inflammatory events (Table 8), and they may either be associated with the invading immune cells or the modulation of inflammatory pathways. Upregulation of miR-223 is associated with the presence of neutrophils [25], [78], [79], which transiently infiltrate the spinal cord early after injury [2]. Sustained upregulation of this microRNA at 3 and 7 dpo could be the result of the persistent presence of T lymphocytes and macrophages in this area [80], although further confirmation of this hypothesis is required. T cell infiltration could also explain the observed upregulation of miR-142 expression, as this is a highly expressed and cell type-specific microRNA in these immune cells [54]. Microglia and monocyte activation upon injury may also be a consequence of the downregulation of miR-124 [81], otherwise a well-known neuronal microRNA [82].

**Table 8:** Inflammation-related microRNAs with significant changes in expression in the present study.

MicroRNA	Expression changes	Targets, effects and references
*Associated with immune cells*		
miR-124	D3, D7	In quiescent microglia [81]
miR-142	U1, U3, U7	In T-cells [54]
miR-223	U1, U3, U7	In neutrophils [151], particularly in SCI [25]
*Pro-inflammatory*		
miR-17-92 cluster (miR-17 & 20)	U3	PTEN [152], TGFBR2, pSMAD2, SMAD4 [92]
hsa-miR-106a	U3	IL10 [94], microRNA not described in rats
miR-124	D3, D7	IêBZ [153]
miR-181b	D7	CYLD [97]
*Anti-inflammatory*		
let7a	D7	IL-6 [85]
miR-9	D3, D7	NF-êB [89], [154]
miR-125b	D7	TNF alpha [86]
miR-146a	U7	TRAF6, IRAK1 promoting NF-êB [155], induced by NF- êB [95]
miR-199	D7	IKK β [90]
miR-15	U3	IKK α [156]
miR-223	U1, U3, U7	IKK α [156]
*Both pro and anti-inflammatory*		
mir-21	U3, U7	PTEN [97] activating NF-κB; PDCD4 [98] inhibiting NF- êB and promoting IL-10 [99], [100]

Expression changes, targets, and references are detailed for the microRNAs associated with immune cells, with pro-inflammatory or anti-inflammatory roles, or with dual roles. Column code abbreviations are the same as in table 6.

In addition to immune cell invasion or activation, the inflammatory response is modulated by a vast number of molecular immune mediators, such as cytokines (TNF-α, IL-6, etc.) chemokines, and growth factors [2], [83]. Key molecules in these inflammatory pathways are known to be targets of microRNAs that underwent changes in expression during our analyses (Table 8), suggesting that microRNAs may contribute to the regulation of the inflammatory response following SCI. For example, the increased levels of pro-inflammatory factors such as TNF-α [84] or IL-6 [8] following injury may result from the reduced expression of their regulators, miR125b and let7a, respectively [85], [86]. These and other pro-inflammatory cytokines released in the injured area [47], [87] induce inflammation through the NF-κB signaling pathway, which is also highly regulated by microRNAs [88]. In fact, our data suggest that a downregulation of miR-9 and miR-199 may contribute to inflammation by reducing the inhibition of NF-κB pathway genes, namely, *p50NFκB* or *ikkβ* [89], [90], [91]. Inflammation may also be induced through the inhibition of anti-inflammatory pathways, such as the inhibition of TGF-β pathway molecules pSMAD2, SMAD4, and TGFBR2 by members of the miR-17-92 microRNA cluster [92] or the silencing of the anti-inflammatory neuroprotective cytokine IL-10 [93] by hsa-miR-106a [94]. Conversely, changes in microRNA expression may reduce the activation of the inflammatory NF-κB Ρpathway; for example, this may have occurred via the decreased expression of miR-124 and miR-181b at 3 and 7 days after injury and the increased expression of miR-15, miR-223 and miR-146a (Table 8). Interestingly, miR-146a upregulation is driven by NF-κB, which in turn is negatively regulated by this microRNA [88], [95]. Thus, overexpression of miR-146a at 7 dpo may be a consequence of an increase in NF-κB on previous days [96], which in turn may have caused the inactivation of this pathway via a negative feedback mechanism. The inflammatory effects of miR-21 are less clear, as they exhibit both pro- and anti-inflammatory activities in the NF-κB pathway. MicroRNA miR-21 targets PTEN, a negative regulator of NF-κB [97], as well as PDCD4, which promotes NF-κB activation and inhibits the expression of IL-10 [98], [99], [100].

Changes in several of these “inflammatory” microRNAs after spinal cord injury have been described in previous studies. Nakanishi *et al.* [5] observed similar changes in miR-223 and miR-124 expression, which were also observed by Liu *et al.* [6], and these studies also identified coincident expression changes in miR-21, miR-146a, and miR-17, among others. These authors proposed that changes in other microRNAs may also modulate inflammation after SCI. In agreement with their results, we observed the downregulation of miR-127, miR-181a, miR-411, miR-99a, miR-34a, miR-30b, and miR-30c, which according to Liu [6] should lead to increased inflammation. On the contrary, other microRNAs identified by these authors, such as miR-152, miR-214, miR-206, and miR-221, either did not show significant expression changes or showed opposite behaviors in our analyses (such as the downregulation of miR-1). However, the putative roles of these microRNAs in inflammation are based on *in silico* predictions and thus lack direct evidence.

Inflammation is directly related to other important processes in SCI pathophysiology, particularly astrogliosis. Several microRNAs involved in inflammation have also been shown to undergo changes in expression following astrocyte activation *in vitro* [101], including the upregulation of miR-146 and the downregulation of miR-455, which is in agreement with our results. According to Sahni *et al.* [102], BMP-induced downregulation of the inflammatory miR-21 causes GFAP overexpression and astrogliosis following SCI. Surprisingly this microRNA clearly appeared to be upregulated after injury in the present study as well as in previous reports [6]. Astrocyte activation also seems to be promoted by the upregulation of miR125b, which leads to GFAP and vimentin overexpression and *cdkn2a* silencing *in vitro* [103]. However, mir-125b demonstrated significant downregulation after injury in the present study. It is possible that the changes in expression for both mir-125b and miR-21 observed in the present study are associated with an infiltration or a response by cell types other than astrocytes. More information is required to determine the precise roles of these microRNAs.

In addition to deleterious processes such as inflammation and cell death, trauma also triggers regenerative processes in the damaged spinal cord. In fact, following injury, the spinal cord displays an overexpression of growth factors (*e.g*., BDNF) and their receptors (*e.g*., trkB), as well as an overexpression of axonal guidance molecules (*e.g*., semaphorin 3) and extracellular matrix proteins (*e.g*., decorin, lumican and collagens) [104], which may promote cell survival and axonal regrowth. Our data provide evidence for the downregulation of the microRNAs miR195 and miR30a, which have been shown to target the growth factor BDNF [105]. According to our IPA networks, the downregulation of miR-183 could also promote BDNF expression (see Figure 7, network 4), whereas the downregulation of miR-329 and miR-331 may induce the overexpression of *sema3*. Moreover, the downregulation of miR-29, which is a modulator of ECM homeostasis [106], may induce the overexpression of key pro-regenerative matrix molecules, such as laminin, collagen, and fibronectin. Thus, the downregulation of each of these microRNAs may contribute to the regenerative processes taking place in the damaged spinal cord by promoting axonal targeting, neuronal survival and neurite outgrowth. However, the pro-regenerative miR-133b, which is a key determinant of the regenerative capability of zebrafish spinal cord neurons [107], also appears to be significantly downregulated following injury.

The profiles that were observed in the present study contrast with those reported from previous studies. Comparing our data to the results of Liu *et al*. [6], there are inconsistencies related to nearly half of the expression changes described by these authors, and this previous study did not observe the global microRNA downregulation following injury that was observed here. These differences are even more striking when our results are compared with those of Nakanishi *et al*. [5]. These authors identified only 5 upregulated and 5 downregulated microRNAs following injury, which is in clear disagreement with the hundreds of downregulated microRNAs that were identified in the current study. There are fewer differences between our study and a recent study by Strickland *et al*. [44], who also observed an extensive microRNA downregulation. Several factors may contribute to these differences. MicroRNAs exhibit species- as well as strain-specific expression patterns [16] that may contribute to the observed differences between studies. However, the comparisons that were made to data from previous studies revealed a general agreement in terms of the spinal cord expression patterns discovered for vertebrates, suggesting that phylogenetic differences do not cause significant changes in microRNA expression. In addition, methodological aspects such as the injury type and severity, the microarray procedures or the data analysis may also contribute to such differences. Leaving aside aspects related to microarray hybridization and data analysis, which are far beyond the scope of this article, the injury model is likely responsible for many of the observed differences in microRNA expression patterns. Severity and injury type strongly determine the pathophysiology and functional outcome of SCI [7], and these are reflected in the gene expression pattern. In fact, according to the data from De Biase and colleagues [7], mild and moderate contusion injuries induce a strong increase in the number of up-regulated genes 7 days after trauma, whereas down-regulated gene expression predominates following severe injury. A similar conclusion was reached by Strickland *et al*. [44], who reported a significant correlation between BBB functional score and the expression levels of specific microRNAs (miR-129-2 and miR-146a). SCI severity has been shown to determine the timing and degree of neutrophil infiltration [108] as well as the expression profile of miR-223 following SCI [25].

Despite extensive disagreement, several microRNAs showed concordant changes in expression across studies; these included the upregulation of miR-223 and miR-21 and the downregulation of miR-124 and miR-219, which were observed in most, if not all, of the examined studies. Moreover, the microRNAs miR-103, miR-107, miR-133a, miR-145, mir146a and miR-98, which presented altered expression at 7 days after SCI in both Liu's study [6] and ours, demonstrated significant alterations in the expression of their targets, according to De Biase *et al*. [7]. The microRNA expression changes that were observed in this study are also in agreement with those predicted from the mRNA data of De Biase and colleagues [7]. These analyses identified four microRNAs with expression changes at 7 dpo; these included miR-340-5p and miR-369-3p, which are both involved in proliferation and adipogenic differentiation, as well as the proapoptotic miR-184 [109] and miR-466b. It may be surprising that only four microRNAs out of the 35 microRNAs inferred from the changes in gene expression levels (see Table 2) are confirmed in the present analysis. However, according to Cheng and Li [14], one of the advantages of the computational method employed is that the expression changes of the target genes for a given microRNA reflects its effective regulatory activity change rather than expression change (since the expression level of a microRNA may not reflect its ability to down-regulate target genes). Thus, microRNA expression levels measured by microRNA microarrays may reflect microRNA abundance but not the actual regulatory activities of the mature microRNAs. However, and taking together the experimental and probabilistic computational approaches, these microRNAs can be highlighted as those playing a confirmed role in this model of spinal cord injury.

In the present study, we evaluated microRNA expression profiles using a rat contusive spinal cord injury model and described a global, progressive downregulation of microRNA expression following SCI, which parallels a previously observed mRNA upregulation. The expression changes identified in this study involved many microRNAs that had previously been reported to fluctuate following SCI, although many discrepancies between studies were also present. The biomathematical analysis of these data allowed us to recognize the implications of many of these microRNAs in the diverse biological processes triggered by SCI. We have described several novel changes in microRNA expression following SCI that require additional analyses for validation and to unravel their functional roles. This study and future analyses will contribute to furthering our knowledge of the mechanisms regulating SCI pathophysiology.

## Materials and Methods

### Ethical Statement

All animal procedures were performed in accordance with the normative R.D. 1201/2005 10-10 from the Spanish Ministry of the Environment and the Agriculture Council of the Castilla-La Mancha animal ethics committees and were approved by the ethical committee at the Hospital Nacional de Paraplejicos. The laboratory animals employed in this study were acquired and cared for in accordance with the guidelines published in the NIH Guide for the Care and Use of Laboratory Animals and the principles presented in the “Guidelines for the Use of Animals in Neuroscience Research” by the Society for Neuroscience. All efforts were made to minimize suffering as well as the number of animals used. The Approval Committee Identification Numbers obtained for the development of this study are 28/2007, 46/2008 and 42/2008.

### Surgical methods and sample collection

Adult, female Wistar rats weighing approximately 200 g were used for all experimental procedures. These animals were divided into three distinct groups: one group without surgery prior to extraction (control group), one group given laminectomies with contusions (injured), and one group given only laminectomies (sham). Injured and sham animals were sacrificed at 1, 3, and 7 days postoperation (dpo). Each group was composed of 5 animals. Animals were anesthetized with intraperitoneal sodium pentobarbital at 40 mg/kg (Dolethal, Vetoquinol, Lure Cedex, France). Contusion was performed at vertebral thoracic level 8 (T8) using an IH Spinal Cord Impactor from Precision System and Instrumentation, LLC, Va, which induced an impact of 200 kilodynes. Medullar fragments 1 cm long, which were centered on the injury, were extracted after the animals were sacrificed. These fragments were maintained in RNAlater buffer (Qiagen) until RNA purification. A second set of animals was treated in parallel to assess histopathology. For this purpose, the animals were anesthetized and transcardially perfused with saline and 4% paraformaldehyde at postoperative time points indicated above. Spinal cord fragments were removed and embedded in O.C.T.™ (Tissue-Tek). Cryosections that were 20 µm thick were stained with Eriochrome/cyanine, according to standard procedures.

### RNA isolation and quality evaluation

Total RNA was extracted using the Qiazol Lysis Reagent (Qiagen, Valen, CA, USA) and purified using the miRNeasy isolation kit (Qiagen). The total RNA concentration was determined by ultraviolet absorbance at 260 nm, and purity was determined with the 260/280 and 260/230 nanometer ratios using a NanoDrop ND 1000 spectrophotometer. RNA integrity was determined according to the electropherogram, and the derived RQI (RNA Quality Indicator) index values were obtained using the Experion microcapillary electrophoresis system (Bio-Rad). Only samples with RQI values over 7.5, *i.e.* those with well-defined electropherograms, and samples with 260/280 and 260/230 ratios between 1.8 and 2.2 were used for the subsequent analyses.

### Microarray hybridization

Total RNA from the 35 animals included in the analysis was hybridized to miRCURY LNA ™ microRNA arrays (Exiqon) containing probes for all microRNAs included in version 11.0 of the Sanger mirBASE. RNA preparation, hybridization, staining, and scanning of the microRNA arrays were performed according to Exiqon protocols. Briefly, 1 µg of total RNA was labeled with Hy3 dye (Exiqon) and hybridized to the miRCURY microarray in a hybridization oven (Shel Lab, Agilent Technologies) with rotation at 20 rpm and 65°C, using Surehyb hybridization chambers (Agilent Technologies). Internal controls were spiked to control for proper hybridization, washing and scanning. The microarrays were scanned, and their signals were analyzed at the Functional Genomics facility of the Scientific Park of Madrid using an Axon GenePix 4000B microarray scanner (Axon Instruments) and the Axon GenePix Pro software with the corresponding GAL files (miRCURY LNA microRNA Array, v.11.0; hsa, mmu & rno, Exiqon). Microarray scanning was performed within the first 6 hours after hybridization to minimize possible bleaching bias. The resulting hybridization values were uploaded to the GEO database (http://www.ncbi.nlm.nih.gov/geo/), where they may be queried under accession number GSE19890. All data are MIAME-compliant.

### Microarray data analysis

Prior to each analysis, quality controls were performed on the arrays images and data. The presence of scratches or staining or hybridization artifacts on the arrays was assessed by visual inspection of the images, which was complemented using background, M, A and M vs. A plots from the marray package in R [110]. Arrays showing significant scratches, dye problems, or bias were discarded, and the samples were reanalyzed using new arrays.

Microarray hybridization data stored as .gpr files by the GenePix software were employed to estimate microRNA expression in each sample. A microRNA was considered to be expressed when its corresponding probes showed hybridization signals 2 standard deviations above the background level. MicroRNAs were excluded from further analyses if they were not considered present in at least 3 individuals from at least one experimental group. Hybridization data were normalized according to the Variance Stabilization Normalization method proposed by Gusev [111] and implemented in the vsn package of R. Because Exiqon microarrays contain 4 replicates for each probe, the median of these four values was used as the estimate of the expression value. The median was employed to minimize the effect of anomalous values on expression estimation.

Global microRNA expression was explored using hierarchical clustering (HC) and the principal component analysis multivariate techniques implemented in the application MeV vs. 4.5.1 from the TM4 microarray software suite [112], [113]. For HC, Euclidean distance metric and average linkage clustering method were employed.

Comparisons of the microRNA expression patterns among groups were performed using both parametric and non-parametric methods. First, the normalized values were filtered using the R package *Genefilter* to eliminate invariant microRNAs, *i.e.*, those with gene expression without an interquartile range above 0.5 for all samples. Parametric comparisons were performed using a paired Student t-test analysis together with a Bayesian inference of variance, according to the methods implemented in the Limma package [114]. The Limma application allowed for the estimation of the fold change and its significance according to the Student t-test and the False Discovery Rate developed for multiple hypothesis testing [115]. Non-parametric comparisons were performed using the Rank Product test implemented in the RankProd (v.2.8.0) package of R.

Comparisons regarding the microRNA changes in expression related to the observed variations in their target mRNAs were performed using lists of differentially expressed microRNAs from the present study, and the mRNA expression profiles were obtained from the supplementary material of De Biase *et al.* [7]. MicroRNA activity was studied for the list of mRNAs that demonstrated significant changes in the injured rats after 1 or 7 days following moderate and mild contusions. The matrix comprising the predicted binding affinity scores for all rat microRNAs, according to the miRanda prediction algorithm, was obtained from the miRNAMap database (http://mirnamap.mbc.nctu.edu.tw/html/downloads.html). We first applied Fisher's Exact Test for each differentially regulated microRNA to determine whether the number of differentially regulated predicted target mRNAs was greater than would be expected by chance (p<0.05). We also evaluated correlations between microRNA and mRNA expression, according to the method developed by Cheng and Li [15]. Statistical calculations for this approach were computed using the C++ winmir program [15], which is available at http://homes.gersteinlab.org/people/cc59/InferMiRNA/infermir.html).

Differentially expressed microRNAs, according to the different comparisons and methods applied, were used to infer their biological functions and generate molecular networks according to the enrichment analyses of Gene Ontology (GO) terms and Ingenuity Pathways (Ingenuity Systems, version 7.6; www.ingenuity.com). GO analysis was conducted using the approach proposed by Gusev [30]. Targets for microRNAs showing significant expression changes were obtained from the miRNAMap database (http://mirnamap.mbc.nctu.edu.tw/html/downloads.html). The resulting list of genes was employed to conduct a Gene Ontology Enrichment Analysis using DAVID bioinformatics resources (http://david.abcc.ncifcrf.gov; see [116]) using all genes in the rat genome as a background. Biological Process and Molecular Function GO terms with FDR-corrected p-values below 0.05 were selected. According to the methods of Gusev [30], these sets of overrepresented GO categories were filtered to select only those that were targeted by 100% or over 50% of the microRNAs in each comparison.

Ingenuity molecular networks and function and disease nets were created based on the data concerning microRNA and mRNA expression and significant expression changes identified by previous analyses. Here, the networks represent a highly interconnected set of molecules derived from the input data set. Biological functions and processes were attributed to networks by mapping the molecules in the network to functions in the Ingenuity ontology and by performing a right-tailed Fisher's exact test to determine the significance (p-value) of any over-representation of proteins for a function compared to the result expected for a random set of proteins. The top-ranked biological functions were those with the lowest p-values.

### Real-Time PCR

TaqMan Real-Time PCR with the 2 ^−ΔΔCt^ relative quantification method was performed using a Taq-Man 7900HT Fast Real-Time PCR system (PE Applied Biosystems). The endogenous control for normalization was U87 RNA, and the calibrator sample was the average of the group controls (no surgery before extraction). TaqMan assays containing primers and TaqMan probes and the TaqMan microRNA Reverse Transcription kit were provided by Applied Biosystems and were used according to the manufacturer's instructions. Briefly, 10 ng of total RNA was used in the reverse transcription reaction, and approximately 2 ng from that reaction was used for subsequent amplification. The Real-Time reaction was performed using 5 samples per experimental group, consisting of those that both were and were not included in the arrays, and each sample was run in triplicate. The reactions were programmed in the 9600 emulation mode, and the steps consisted of one cycle for 10 min at 95°C, 40 cycles for 15 sec at 95°C, and one final cycle for 60 seconds at 60°C.

## Supporting Information

File S1 **Excel workbook containing raw, vsn normalized and filtering microRNA expression data.** The excel workbook contains 4 worksheets. First worksheet details the untreated hybridization raw data for all probes in the array. The first 4 columns detail the position information of the probes in the array (ID: identity number, Blk: array block, Col: vertical coordinate within block, Row: horizontal coordinate within block). Each individual is coded as its experimental group X individual number. The second worksheet details the VSN normalized values and the criteria used to filter undetected and invariant data. Array and individual codes are represented as in previous spreadsheet. Filter codes indicate D.F.: detection filter (yes = detected, no = non-detected); IQR>0.5: variability filter (yes = probes showing more than 0.5 interquartilic range across all individuals, no = probes with IQR below 0.5); Both: probes that pass both filters (yes) or not (no). The third worksheet shows the vsn normalized values for all probes passing both filters. The forth worksheet shows the vsn normalized data for all detected and variant microRNA after computing the median value of the repeated probes for each microRNA.
(XLS)

File S2 **Excel worksheet detailing the loadings for each microRNA expression value in the 3 first components obtained after Principal Component Analysis.** Analyzed data correspond to the vsn normalized expression values of the 463 microRNA showing detectable and variable expression.
(XLS)

File S3 **MicroRNA expression changes after spinal cord injury.** Excel workbook detailing the changes in microRNA expression after SCI according to parametric (t-student plus FDR adjustment) and non-parametric (Rank Product) methods. The **first worksheet** shows all significant expression changes of the analyzed microRNAs and comparisons (LS: injury, SH: sham, CT: control). Comparisons as detailed in figure 3A. The **second worksheet** shows the microRNAs showing significant changes in different comparisons according to both parametric and non-parametric tests.
(XLS)

File S4 **Excel worksheet comparing the data of the microRNA with the highest expression levels according to our studies and from previous studies on vertebrate microRNAs** [**6]**, [17], [18], [19], [20], [21], [22****] **.** Data from the present analysis are shown in columns 1 to 9. Columns 2 to 8 indicate the microRNA with the highest expression in control, sham and injured animals of the present study. Column 9 indicates the detection (P) or not (A) of the different microRNA in our microarray analysis.
(XLS)

File S5 **Excel worksheet detailing the enriched GO terms targeted by miRNA with significant expression changes across comparisons.** The first worksheet (Enriched GO terms) details the significantly enriched GO terms for all comparisons. The table details the probability (p) and the number of miRNAs targeting each GO term respect to the total number of miRNAs targeting GO terms in a particular comparison (#M). The second worksheet (percentages matrix) shows the percentage matrix employed in the cluster analyses. Percentages correspond to the percentage of the total number of miRNA with significant expression changes in a given comparison targeting a specific GOterm. The third worksheet (clusters) details the GO terms in the different clusters according to the hierarchical clustering analyses (codes A to M correspond to the cluster identified in figure 6).
(XLS)

File S6 **Excel worksheet detailing the most relevant networks associated to the present microRNA expression changes according to IPA analysis.** First column details the groups under comparison, second column describes the functions associated to each molecular network; score denotes the value of the IPA score (similar to the fisher exact test); and the last column detail the set of molecules included in each network. These networks are depicted individually in distinct excel sheet. Networks with comparisons in bold type are illustrated in figure 7.
(XLS)
